# Effects of iron deficiency and iron supplementation at the host-microbiota interface: Could a piglet model unravel complexities of the underlying mechanisms?

**DOI:** 10.3389/fnut.2022.927754

**Published:** 2022-10-04

**Authors:** Munawar Abbas, Zeynep Hayirli, Hal Drakesmith, Simon C. Andrews, Marie C. Lewis

**Affiliations:** ^1^Food and Nutritional Sciences, University of Reading, Reading, United Kingdom; ^2^MRC Human Immunology Unit, MRC Weatherall Institute of Molecular Medicine, University of Oxford, Oxford, United Kingdom; ^3^School of Biological Sciences, University of Reading, Reading, United Kingdom

**Keywords:** iron deficiency, enteric infection, iron supplementation, neonatal gut health, anaemia, immunity, gut microbiota, dysbiosis

## Abstract

Iron deficiency is the most prevalent human micronutrient deficiency, disrupting the physiological development of millions of infants and children. Oral iron supplementation is used to address iron-deficiency anemia and reduce associated stunting but can promote infection risk since restriction of iron availability serves as an innate immune mechanism against invading pathogens. Raised iron availability is associated with an increase in enteric pathogens, especially Enterobacteriaceae species, accompanied by reductions in beneficial bacteria such as Bifidobacteria and lactobacilli and may skew the pattern of gut microbiota development. Since the gut microbiota is the primary driver of immune development, deviations from normal patterns of bacterial succession in early life can have long-term implications for immune functionality. There is a paucity of knowledge regarding how both iron deficiency and luminal iron availability affect gut microbiota development, or the subsequent impact on immunity, which are likely to be contributors to the increased risk of infection. Piglets are naturally iron deficient. This is largely due to their low iron endowments at birth (primarily due to large litter sizes), and their rapid growth combined with the low iron levels in sow milk. Thus, piglets consistently become iron deficient within days of birth which rapidly progresses to anemia in the absence of iron supplementation. Moreover, like humans, pigs are omnivorous and share many characteristics of human gut physiology, microbiota and immunity. In addition, their precocial nature permits early maternal separation, individual housing, and tight control of nutritional intake. Here, we highlight the advantages of piglets as valuable and highly relevant models for human infants in promoting understanding of how early iron status impacts physiological development. We also indicate how piglets offer potential to unravel the complexities of microbiota-immune responses during iron deficiency and in response to iron supplementation, and the link between these and increased risk of infectious disease.

## Introduction

Inadequate nutrition is recognized as a major factor in anomalous development during early life and has serious implications for long-term health (1–3). Iron is an essential nutrient since it acts as a key prosthetic component (e.g., for haem and iron-sulfur clusters) associated with proteins that have vital roles in a wide range of key biochemical processes such as oxygen transport, the respiratory chain and the Krebs cycle. Iron deficiency (ID), where body iron stores are becoming depleted occurs when iron requirements exceed intake. As these store become exhausted, iron deficiency anemia (IDA) develops and affects around 1.2 billion people worldwide (4, 5). IDA is defined as serum hemoglobin (Hb) levels of < 105 and < 100 g/L in 4 and 9 month old infants, respectively (6). Those at particular risk of ID are the under-fives and pregnant women from low- and middle-income countries (7). In full-term infants born to iron-sufficient mothers, transplacental delivery of iron results in high neonatal reserves, around 75 mg/kg, primarily as hemoglobin and in the form of ferritin and hemosiderin (8, 9). Following birth, excess iron, derived from hemoglobin, is immediately transferred to storage compartments and consequently, under normal conditions, iron supplied through the diet is not required during the first 4–6 months of life (10). This may partly explain why human breastmilk possesses such a low iron content (∼0.35 mg/L; bioavailability of 45–100%) (11). However, after ∼6 months, infant iron stores derived from the mother become depleted whilst the developmental demand for iron increases due to higher erythropoietic and brain activity, along with increased tissue accretion as a result of high growth rate (12, 13). Demand for iron soon exceeds that available from breast milk prompting the need for iron from complementary foods and/or supplementation (14).

Dietary iron requirements during pregnancy are significantly increased. Based on a pre-pregnancy weight of 55 kg, it is estimated that an additional 1 g of iron is required during pregnancy, equating to around 3.6 mg/day on average (15). This is due to increasingly higher demands from the fetus and placenta, and rapid expansion of the maternal vascular volume during the latter half of gestation especially (16). However, in both poor and affluent societies a large proportion of women enter pregnancy with ID or IDA (17) since around 50% of women of childbearing age in low and middle income countries are anemic, although there is significant regional variation (18). In both ID and IDA, the iron endowment that new-borns receive from their mothers is often reduced such that iron reserves are depleted well before 4–6 months (when weaning generally commences) leading to the rapid onset of IDA. Infants born to anemic or iron-deficient mothers, and those with low birth weight, begin life with reduced iron stores and are at higher risk of developing ID before 4–6 months (19, 20). Preterm infants are also at increased risk of developing ID as maternal iron transfer to the fetus mostly occurs during the final trimester (21).

Rapid erythropoiesis, inadequate dietary iron consumption and limited iron bioavailability (linked to reduced absorption following enteric infection and/or dietary inhibitors) all contribute to increased risk for ID throughout infancy and childhood. At this time, iron requirements are high due to rapid growth (22). School-age children primarily consuming unfortified cereal-based diets are at greater risk of ID owing to low dietary iron intake (23). In addition, non-haem iron, the form derived from plant sources, has lower bioavailability and is more sensitive to enhancers (e.g., ascorbic acid) and inhibitors (e.g., phytate) of iron absorption compared to haem iron, the form derived from animal sources.

The iron demands of fully functioning adult immune systems are high and consequences of ID include the inhibition of neutrophil function, reduced microbicidal ability of macrophages, reductions in T-cell numbers and thymic atrophy (24–27). However, the mechanisms underlying such effects are poorly characterized and even less is understood regarding the impact of ID on the rapidly developing immunological architecture and immune-associated cell population expansion during infancy. These are important aspects of childhood IDA since the functionality of the adult immune system is highly dependent on appropriate development in early life. This indicates that childhood IDA could have longer-term detrimental consequences on immune function, even if iron-sufficiency is subsequently achieved in adulthood. It is now recognized that even in the absence of IDA, dietary ID has adverse functional consequences and is detrimental to many areas of growth during infancy, as previously reviewed (28).

The WHO report on “Daily iron supplementation” endorses iron supplementation as a public-health intervention for infants and children aged 6–23 months across the globe, where the prevalence of anemia in this age group is high (>40%). Recommendations are for daily doses of 10–12.5 mg elemental iron for 3 consecutive months per year for infants and children aged 6–23 months, which increases to 30 mg for preschool-aged children (29). However, a recent placebo-controlled study of iron supplementation for 12 weeks in ∼8 month old Bangladeshi infants concluded that although rates of anemia were reduced, there was no effect on developmental functional outcomes, including cognitive ability (30). An important caveat to iron supplementation recommendations is in areas with endemic malaria where iron supplementation is only recommended to those infants with access to malaria-prevention strategies (30). This follows comprehensive meta-analyses which concluded that there is a possible link between iron supplementation and increased risk of mortality or hospitalization from malaria (31).

## Iron deficiency and infection risk—A trade-off

Complex relationships exist between anemia and infection. ID may result in increased infection morbidity, presumably due (in part at least) to inadequate iron supply to the immune system (32). However, ID can also protect against infection (33) in both humans and animal models. Since iron is crucial for bacterial growth, in particular for pathogens such as *Escherichia coli* (34, 35), it follows that insufficient iron availability, achieved by nutritional immunity mechanisms in the host, is an effective strategy to limit pathogenic growth and thus reduce infection risk (36). Consistent with this, various *in vitro* studies have reported reduced growth of potential enteropathogens, including *Campylobacter jejuni* and *E. coli*, within iron-deprived environments (37, 38). For example, limiting iron availability resulted in reductions in *E. coli* abundance to 0.8% compared to 10.7% in an iron-sufficient controls. Non-nutritional factors, such as infection and inflammation, also influence iron metabolism and can cause “anemia of inflammation,” previously termed anemia of chronic disease. This response is instigated by the host to limit systemic iron availability and thus combat ongoing infection (39), and is predominantly mediated through the upregulation of hepcidin, as we have previously reviewed (25). This peptide hormone acts as a major regulator of iron homeostasis. Hepcidin is secreted primarily by hepatocytes in response to various factors including body iron stores and plasma iron levels. It controls iron absorption, recycling, and release from stores by binding to the cellular iron exporter, ferroportin, causing it to internalize, leading to iron retention within cells. This consequently limits iron availability to extra-cellular pathogens (40, 41). Hepcidin expression is also responsive to elevated inflammatory cytokines including IL-6 and IL-22 (33, 42). Therefore, anemia of inflammation is characterized by adequate or high iron stores but low serum iron (bound to the serum iron chaperone, transferrin) (42). In contrast to ID, anemia of inflammation cannot be prevented or resolved by iron supplementation and may even be intensified by increased dietary iron (40).

To mitigate the increased risk of enteric infection driven by oral iron supplementation, perhaps an alternative route of iron administration could be considered. Intravenous (IV) iron sucrose can be administered to children with ID and is especially useful for those who have failed to respond to oral iron supplementation, for example those who suffer from iron malabsorption due to short bowel syndrome or anemia of inflammation. A further advantage of this IV route is that it bypasses the hepcidin-ferroportin pathway that controls iron absorption in the gut. Such infusions can mitigate ID following 1–5 doses of between 25 and 500 mg for up to 6 months following treatment and appears to be safe, effective and well tolerated (43, 44). However, infrequent, high doses are linked with an increased risk of transferrin oversaturation, although to a lesser extent than earlier non-sucrose-based formulations (45). Although promising at this stage, most of the trials exploring IV iron administration have focused on adolescence and adult populations and it is questionable as to whether it is feasible for young infants in rural settings in Low and Middle-income countries where ID is prevalent.

## Role of iron in the development of the gut microbiota

Bacterial numbers and microbiota diversity increase in relation to distance from the stomach with the largest, most diverse population residing in the colon due to relatively more favorable conditions (46). Due to the influence of several factors, including pH and the chemical form of iron present, predicting the bioavailability of iron to the microbiota for each of the various sections of the intestinal tract remains problematic. However, it is estimated that on average ∼85% of dietary iron remains unabsorbed and colonic iron concentrations are in the region of 25 mM, of which approximately 0.4 mM is in the form of readily absorbable Fe^2+^ (47).

**FIGURE 1:**
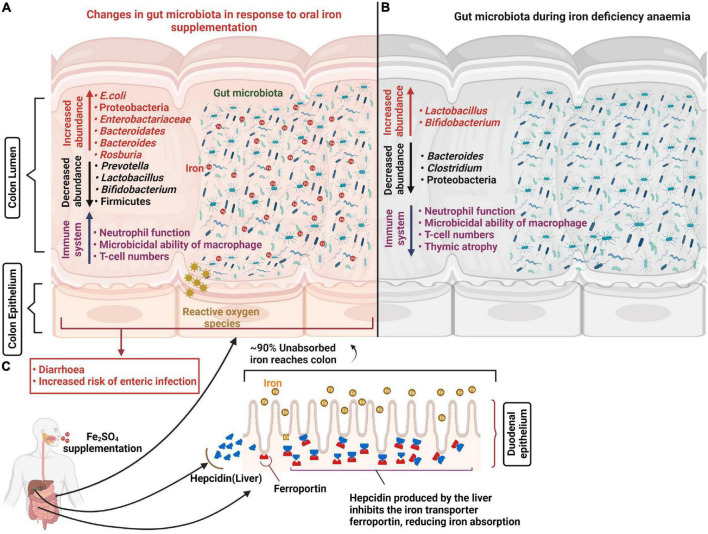
Changes in the gut microbiota in response to oral iron supplementation and anemia. Iron supplementation prevents iron-deficiency anemia and aids the generation of appropriate immune responses to invading pathogens. However, approximately 90% of unabsorbed iron reaches the colon where most bacteria have substantial iron requirements for growth. This excess iron results in decreased abundance of beneficial bacteria including lactobacilli and bifidobacteria and increased abundance of potential pathogens including *E. coli* which increase the risk of diarrhea and enteric infection. In addition, oral iron supplementation may result in the generation of reactive oxygen species (ROS) causing oxidative stress and epithelial damage **(A)**. Abundance of potential pathogens including *Bacteroides* and *Clostridium* decrease in response to iron deficiency anemia and promote beneficial bacteria population growth **(B)**. Hepatic cells release hepcidin in response to oral iron supplementation which internalizes the ferroportin (iron transport protein), reducing iron absorption in the duodenum **(C)**.

The majority of gut bacteria have essential requirements for iron and thus require its availability in the gut. However, the Lactobacillaceae are considered to be iron-independent members of the gut microbiota and preferentially utilize manganese instead (48). It is likely that iron availability influences microbial succession and the developmental stability of the longer-term microbiota. Increased iron availability has also been associated with a raised risk of the presence, or virulence potential, of enteric pathogens including *Salmonella typhimurium*, *E. coli* and Enterobacteriaceae, along with reductions in more beneficial bacteria such as bifidobacteria (49–51). Gut fermentation models inoculated with fecal matter from a child and propagated under iron limited conditions (1.56 ± 0.1 mg Fe L^–1^) showed a relative reduction in *Roseburia* spp., *Eubacterium rectale*, *Clostridium* cluster IV members and *Bacteroides* spp. along with relative increases in *Lactobacillus* spp. and Enterobacteriaceae compared to iron sufficient controls (52). Similarly a recent pilot study assessed the composition of the microbiota in IDA infants and young children concluding that ID is associated with distinct microbial signatures with increased abundance of Enterobacteriacea and Veillonellaceae, and decreased Coriobacteriaceae relative to healthy non-ID controls (53). Consistent with this, several large-scale human trials using various doses for different durations (2–18 months) have, in general, reported that oral iron supplementation is associated with higher risk (1–23%) of developing diarrhea in infants (54–56). However, the outcomes of such studies have been inconsistent (57, 58), which suggests the link is complex and that perhaps other factors are at play. One theory is that the effects of iron supplementation on the risks of enteric infections are highly dependent on the composition and metabolic activity of the underlying microbiota (59), which in turn is influenced by iron availability during the earlier developmental stages. These studies largely reported compositional changes to the microbiota during ID and IDA (Figure 1). However, an important consideration is how sustained these effects may be once sufficient iron levels have been attained. A recent early-life piglet study showed that ID (rapidly progressing to IDA during the trial) reported 27 bacterial genera differences in feces following 32 days of iron restriction (from 2 day of age) compared to iron-sufficient controls. It went on to demonstrate that when both groups received oral iron supplementation (standard 180–300 mg Fe/kg of diet) in weaner mix for a further 30 days, no differences in either bacterial populations or bacterial products of fermentation (volatile fatty acids) were detected. Although normal microbial communities appeared to have been be restored following this period of ID and IDA, bacterial colonization and succession in the gut is the primary driver of immune development which occurs in a programmed and sequential manner and is largely complete by 49 days following birth (60), well before the completion of the trial. Therefore different patterns of microbial colonization driven by iron limiting conditions in the gut is highly likely to have had considerable impact on immune function in later life and could effect susceptibility to infection. In addition, this study reported findings from limited pigs (∼*n* = 10/treatment group). Being outbred there are considerable inter-litter and inter-individual variations and in the absence of litter-matching to accommodate this, treatment differences in gut microbiotas at 61 days resulting from earlier ID/IDA would have to have been larger than physiological differences between piglets to have been observed. In addition, the trial was completed using 2 replicate groups and we have previously demonstrated that minor environmental variations during the first day of life exerted sustained influences on both the microbiota and metabolic phenotype which were of a higher magnitude then differences linked to divergent nutrition (61). Taken together, this suggests that important questions of longer-term impacts of early-life ID and IDA on the gut microbiota, and wider development, remain unanswered.

Given the importance of the early microbiota for immune development (62) and susceptibility to infectious disease (63, 64), and the prevalence of global infant ID and IDA, it is perhaps surprising that early bacterial colonization and succession in the infant gut under such conditions have not been explored in more detail. Understanding the underlying mechanism, and the links with the early iron-deficient microbiota and developing immune systems could enable the development of improved approaches for reducing the burden of ID and IDA whilst avoiding the unwelcome side-effects of oral-iron supplements including epigastric comfort, nausea, vomiting (65) and the increased risk of infection. So far, studies have tended to focus directly on the response of the gut microbiota and immune development following iron supplementation in ID and IDA infants, rather than underlying mechanisms.

## Iron and gut-associated immune system development

Immune systems are immature at birth and along with considerable thymic activity (66), the gut is a principal location for immune development in infants. This is because exposure to resident microbial populations (and to a lesser extent non-microbially derived ingested antigens) is the primary driver of both adaptive and innate immune-associated cell proliferation, education, and expansion. The gut microbiota is both maternally- (67) and environmentally acquired (68–70) and develops rapidly postnatally and, as highlighted above, the vast majority of these microbes have fundamental requirements for iron. The composition of the microbiota plays a key role in reducing enteric infection rates by competing for nutrients and inhibiting the colonization and proliferation of pathogenic organisms. In addition, the pattern of microbial succession in the gut is a key factor in the development of the microbiota (69–72) and variations from normal patterns of sequential colonization can alter the developmental trajectory of both the microbiota and immune system, as demonstrated by exposure to antibiotics during early life (73). Thus, luminal iron availability is likely to have major impacts on pathogen proliferation in the gut directly, through modifying the composition of resident gut microbiota population (which could promote or inhibit pathogen colonization and expansion), and also through changes in the development of immune function.

The B-cell population is one of the key players in microbiota homeostasis as it leads to abundant production of secretory immunoglobulin A (IgA), the dominant Ig in the gut, antibodies which recognize commensal bacteria (74, 75). Gut commensals, in addition to shaping B cells present in the gut, may also provide the control points to step down autoreactivity from the systemic B cell population (76). The association between gut microbiota and intestinal IgA is mutualistic, in that diverse and selective IgA production contributes to the development and stability of the gut microbiota which in turn promotes the development of regulatory T-cells supporting homeostatic IgA responses. In addition, gut bacteria influence T-cell proliferation and drive differential development of T-helper (T_*h*_) (77), T-regulatory (T_*reg*_) (78) and memory T-cell populations (79, 80). Secretary IgA is manufactured in both T-cell dependent and independent manners, therefore changes to T-cell populations can influence IgA production. Given the complex interactions between immunity and the microbiota, it follows that anemia- and oral iron-induced changes to intestinal bacterial succession in early-life have the potential to impact immune development, although mechanisms remain unclear.

Iron deficiency during later childhood (< 15 years) has been shown to significantly reduce CD4^+^ T-cell numbers and decrease CD4:CD8 T-cell ratios without impacting IgA, IgM or IgG levels (81). A further study in children found no reduction in CD4:CD8 T-cell ratios, IgA, or IgM levels in response to IDA, but did report significantly lower IgG levels along with reductions in systemic IL-6, and both macrophage and neutrophil phagocytic function (82). These data arise from small scale, limited and contradictory studies in children, rather than infants, and highlight the paucity of knowledge regarding the impact of ID and IDA during the crucial, early stages of immune development. However, a study of immune responses to vaccination in Tanzanian, Mozambican and Dutch children under 5 years of age reported correlations between anemia and lower frequencies of recent thymic emigrant T-cells, isotype-switched memory B-cells and plasmablasts, and showed that modulating iron bioavailability *in vitro* could recapitulate B-cell defects (83). However, this was in the absence of *in vivo* trials where the gut microbiota is likely to have been modified by luminal iron availability and therefore immune development is also likely to be affected. In summary, despite its clear importance (especially when considering the global prevalence of ID, IDA and oral iron supplementation), the relationship between anemia, oral iron supplementation, the microbiota and immune development remains relatively uncharacterized.

## Chemical kinetics and absorption of iron

Dietary iron mostly occurs in the oxidized, ferric (Fe^3+^) form which is poorly absorbed. For iron absorption to occur, it must either undergo conversion to the ferrous (Fe^2+^) ionic state, or be present as haem or in nanoparticulate form (e.g., ferritin cores) (84, 85). A combination of stomach acidity and reducing agents, such as ascorbic acid and ferric reductase enzymes (including duodenal cytochrome B; DcytB) results in reduction of Fe^3+^ to Fe^2+^. Consequently, dietary non-haem iron is mainly absorbed across the duodenum and proximal jejunum in the highly soluble ferrous form *via* the divalent metal cation transporter 1 (DMT1, also known as NRAMP2) (86, 87). To combat poor absorption and gastrointestinal issues associated with oral iron supplementation, an innovative nano iron supplement is currently being trialed in Gambian children (age 6–35 months). Here, the iron supplement, hydroxide adipate tartrate (HAT) remains in a nano particulate form in the gut which may permit adsorption with fewer symptoms (88).

Partial absorption of dietary iron contained in food and supplements is a persistent and considerable obstacle in the treatment of ID and IDA. Typically, oral iron salt absorption varies between 2 and 13% under normal conditions but can be improved, to some extent, to between 5 and 28% if administered following fasting (89). However, a recent randomized, single-blind, crossover study in ID women demonstrated that coadministration of 15 g of galacto-oligosaccharides (GOS) or fructo-oligosaccharides (FOS) and a single 100 mg Fe tablet, labeled with 4 mg ^57^Fe or ^58^Fe, resulted in 45 and 51% increases in absorption, respectively compared to counterparts who received carbohydrate-based placebos (90). Similarly, a 4 week study in iron-sufficient adult Sprague-Dawley rats demonstrated an 8% increase in iron adsorption from standard feed when fortified (5/100 g feed) with GOS (91). The disparities in efficacy between these human and rodent trials could be linked with iron status, or species differences. Some probiotic strains have also been demonstrated to improve iron uptake, as recently reviewed by Rusu et al. (92). For example, meta-analysis of 15 studies found significant improvements in iron uptake in response to administration of *Lactobacillus plantarum 299v (Lp299v)*, but not for other probiotic lactobacilli strains (93). The mechanism appears to be largely *via* probiotic-induced reduction of luminal Fe^3+^ to Fe^2+^ and thus promotion of iron uptake by enterocytes (92).

Since both GOS and FOS are established prebiotics known to selectively stimulate growth and activity of beneficial microbial strains residing in the gut (94, 95), there is potential for both to reduce the impact of increased luminal iron availability on microbial population skewing to a less beneficial phenotype. This could be especially relevant for the Lactobacillaceae which are abundant in healthy infant guts (96). This is because, as previously mentioned, they do not have requirements for iron and so are disadvantaged under iron-rich conditions. However, further research is required to determine whether such prebiotics increase iron absorption in infants and young children, and what effect GOS/FOS-iron combinations may have on the composition and metabolic activity of the gut microbiota during the critical stage of early-life development of other physiological systems. In addition to enhancing iron status, *Lp299v* (along with several other lactobacilli) has been shown to reduce the incidence and/or severity of a range of enteric infections, and to prevent *E. coli* attachment to enterocytes and the associated tight cell junction disruption in Caco-2 monolayers (97–100). Given that oral iron availability is also associated with increased enteric infection, such probiotics may also be beneficial in limiting some of the side-effects associated with oral iron supplementation.

## Dietary iron negatively impacts host-pathogen competition

The universal iron supplementation policy in areas with high prevenance of ID and IDA results in the delivery of additional oral iron to significant numbers of children who are not iron deficient. This is also the case in more affluent societies where infant formula milk is consistently fortified with iron. This “additional” oral iron may ultimately result in higher quantities of luminal iron being made available to the developing gut microbiota (25). Importantly, an *ex vivo* study reported considerably raised growth of bacteria (including *E. coli*, *Salmonella* and *Staphylococcus epidermis*) in the serum of subjects receiving dietary iron supplementation (2 mg of iron/kg body weight). A strong correlation was observed between transferrin saturation and bacterial growth rates (101). This suggests that even modest levels of oral iron supplementation may contribute to bacteremia and may have implications for iron administered intravenously. Furthermore, a randomized controlled study in iron deficient and/or anemic Kenyan infants demonstrated that iron supplementation caused a deleterious shift in the gut microbial profile which included an increase in pathogenic bacterial population levels and decreases in beneficial lactobacillus and bifidobacterial numbers (59, 102). Consistent with this, a study in Swedish infants (non-anemic) demonstrated that the intake of infant formula high in iron (6.6 mg/day) was linked with a relatively lower population of bifidobacteria than counterparts consuming low iron formula (1.2 mg/day). Similarly, a reduction in the abundance of lactobacilli was observed in response to the administration of oral iron drops (6.6 mg/day) along with an increased prevalence of bacterial infection (103). Even modest levels of iron supplementation, equivalent to that received by infants in fortified formula milk (150 mg Fe/day) and substantially below levels considered toxic, was associated with significant changes in the gut microbiota in young rats. This excess iron was also associated with increased 3-hydroxybutyrate and decreased amino acids, urea and *myo*-inositol. These parameters were linked with adverse cognitive development quantified by memory and learning scoring using the passive avoidance test (104). Taking the above together, the results suggest that although oral iron supplementation is adequate in preventing IDA, it also causes a detrimental shift in bacterial populations which is likely to have long-term effects on microbiota and immune system development, and possibly cognitive function. Thus, there appears to be a health trade-off whereby treatment with oral iron supplements can have deleterious as well as beneficial consequences.

## The piglet model of iron deficiency

**FIGURE 2:**
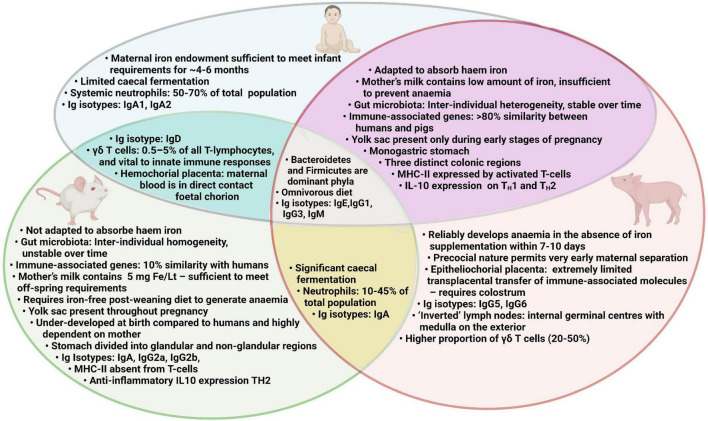
Comparison of human, pig and mouse characteristics relevant to assessing the effect of iron deficiency anemia and iron supplementation.

A suitable animal model would be invaluable in generating mechanistic understanding of the multilateral interactions which occur between the gut microbiota, immune system and iron status. Such a model may also be instrumental in unraveling the reasons why oral iron supplementation may increase the risk of infection and thus aid the development of novel ID treatment strategies to limit such side effects. Rodent models have generated important biomedical information in this field and have several advantages over other animal species including transgenicity, rapid generation time, and accessibility of targeted reagents (105, 106). However, there are important disadvantages to using rodent models for ID which require iron-deficient diets over long periods of time, which is problematic for modeling early-life ID and IDA (107). Furthermore, mice have not adapted to adsorb haem which could limit the translational potential of some studies (107). A summary of human, pig and mouse characteristics relevant to assessing the effect of iron deficiency anemia and iron supplementation is presented in Figure 2.

In contrast, in the absence of iron supplementation neonatal piglets start to become iron deficient within the first week of life. Since early-life environmental and dietary factors can have sustained impact on physiological development (73, 108), it is highly desirable that maternal influence is limited from a very young age and environmental factors are tightly controlled which is far less challenging in precocial species such as the pig; rodents are born relatively underdeveloped and are therefore highly reliant on their mothers during infancy. Consequently, the litter is often the unit which doesn’t conform to the 3Rs (replacement, reduction, and refinement) required by UK legislation, to reduce the number of animals required to generate power. In addition an outbred, rather than inbred, model better reflects the human population, We propose that the piglet model fulfills these criteria, and we provide evidence below to support this.

It is well established that omnivorous pigs are a valuable and tractable model for humans (109) as they share several key features including gastrointestinal immunology, physiology, microbiology, pathologies and dietary requirements (110–114). Full genome studies show that there are fewer difference between pigs and humans, than rodents and humans (115, 116). These factors suggest that pigs are valuable intermediates between highly reductionist, mechanistic studies in rodents, and epidemiological studies and clinical trials in humans. Pup-in-a-cup trials, where rats can be individually accommodated from 5 days of age, have been useful for assessing the impact of early nutrition on physiological development. However, precocial piglets are especially valuable models for early life since their self-sufficiency permits very early separation from their mothers and individual housing within a few hours of birth, thus limiting the maternal influence at this critical period of developmental plasticity. Comparative assessment of pig, mouse and human genomes demonstrated that structural and functional analyses of murine genes involved in immunity an inflammation shared only 10% similarity with humans for measured parameters, whereas in pigs this figure is > 80% (117, 118), Key similarities include the intra-epithelial lymphocytes, and a majority of cytotoxic suppressor T-cells and fewer T_*H*_ cells (119). In pigs and humans, gut microbiotas are considerably more stable over the passage of time than in rodent models. Additionally, the intra-individual variability is reduced in mice compared to that of humans and pigs (120). Generally, the microbiota of pigs and humans also share similar diversities and dominant phyla, including Firmicutes and Bacteroides (121, 122). For these reasons, there is increased potential for determining the mechanisms underlying early microbiota-host interactions in human infants using piglet rather than rodent pup models.

As food animals, there is wide public acceptance of piglet use in research, which can be problematic for other non-rodent species such as primates, dogs and horses. Piglets are particularly prone to ID and will consistently and rapidly develop anemia if iron supplements are not provided (123, 124). Indeed, iron deficiency has been an established issue in the pig industry since the early twentieth century (125) when oral administration of iron salts, as a preventive measure, was first proposed. However, today an early intra-muscular injection of 200 mg of iron is standard husbandry practice throughout the pig industry to prevent the early onset of ID/IDA (126). Piglets are born with very low iron reserves (35–50 mg) which are only sufficient for 3–4 days since daily iron requirements range from 7 to 16 mg (127). Serum iron at day 4 after birth reduces by 5 fold in non-supplemented piglets and is barely detectable after 6 days (128). The situation is exacerbated by the rapid increase in litter size over recent years from 12 to 16 piglets (129) thus placing further iron demands on sows. Therefore, the sow-piglet dyad provides a highly useful potential model in the exploration of how manipulation of maternal feed practices and other interventions may improve iron status in offspring.

Poor efficiency of iron transfer through the placenta is an important contributing factor for the relatively low maternal iron endowment received prior to birth in both humans (130) and pigs (131). In the study by Colomer et al. (132), 156 infants were closely monitored during their first postnatal year. The risk of developing anemia was increased by 6.57-fold in infants born to mothers with anemia (<12 ng/ml) at the time of delivery. The “perfect parasite” is a phrase often used due to the misconception that the fetus is capable of procuring enough iron irrespective of the mother’s iron status. Although iron is transferred to fetal piglets during gestation, iron supplementation in sows during pregnancy leads to only limited improvements in iron status in offspring and is insufficient to combat the development of IDA in piglets (133). Similarly in anemic humans, while iron supplementation during pregnancy improves maternal iron status and may improve pregnancy outcome, including birth weight and reductions in pre-term births, brief periods of iron supplementation are unlikely to counter anemia in off-spring. Increasing the iron endowment received by infants probably requires improved maternal iron status before the pregnancy begins (134).

A further factor contributing to the development of ID in piglets is the relatively low iron content in sow milk (0.2–0.4 mg per L) (135). From this piglets can absorb ∼60–90% resulting in around 1 mg of iron per day which is insufficient to prevent ID in suckling piglets (133). This is similar to humans where breast milk contains around 0.4 mg/Lt (136). However, there is a remarkable capacity for transfer of serum iron to milk in rodents resulting in concentrations of ∼5 mg/L, sufficient to sustain off-spring iron status before weaning (137). Moreover, piglets have the highest growth rates of livestock animals typically increasing their plasma volume by 30% as well as doubling their weight in the first week of life (138) followed by a 10-fold increase from birth weight over the following 5 weeks (139). Most of the functionally active iron (60%) resides in the form of hemoglobin and the majority of the remainder is required for adequate enzymic function and the generation of myoglobin (127). Liver iron stores and sow milk together cannot meet such high iron requirement of piglets.

Although there are numerous physiological similarities between humans and pigs it is important to consider the differences in placentation and other anatomical features. In humans, placentation is haemochorial; maternal blood is in direct contact with the fetal chorion and thus transfer of passive immunity (and other maternal factors) occurs during gestation (140). However, pigs have an epitheliochorial placenta resulting in extremely limited transplacental transfer of immunoglobulins, leukocytes and various T-lymphocyte subsets during gestation (141, 142). For this reason, the neonatal piglet depends almost entirely on passive transfer from colostrum and, to a degree, subsequent milk. Another immunological difference between humans and pigs is lymph node structure. In comparison to humans, pig lymph nodes are inverted with internally placed germinal centers, a medulla located on the exterior and the afferent lymph diffusing to the periphery from the center, although the functional consequences remain unclear (143). Moreover, jejunal Peyer’s patches (JPP) and ileal Peyer’s patches (IPP) occur as multiple isolated follicles in humans, however, in pigs, IPP are continuous structures, but JPP present as isolated follicles. This may suggest functional differences but this is also unclear (144).

In conclusion, here we provide evidence promoting the piglet model as a valuable tool for the provision of novel insight into the mechanisms underlying host-microbe interactions during iron deficiency and in response to oral iron supplementation, especially during early-life. Given the global prevelance of iron deficiency-driven oral iron supplementation, there is an urgent need to identify alternatives to the current strategy (or refine those already used) since this has been correlated with increased risk of infection in already vulnerable infants and children. Exploration of this dilema using traditional model species suffers from challenging constraints that can be overcome within the piglet model.

## Author contributions

MA, ML, and ZH conceived the idea. MA and ML performed literature review, composed the manuscript, and designed the figures. ZH, HD, and SA edited and provided critical feedback and expertise on iron biology. ML was responsible for the overall direction of the manuscript. All authors contributed to the article and approved the submitted version.
